# Voice quality after open partial horizontal laryngectomy vs. total laryngectomy with voice prosthesis: a comparative study

**DOI:** 10.1007/s00405-024-08735-5

**Published:** 2024-07-24

**Authors:** Cecilia Botti, Cecilia Lotto, Paolo Tesauro, Monica Guidotti, Aurora Borghi, Gabriele Molteni, Livio Presutti, Ignacio Javier Fernandez

**Affiliations:** 1 Department of Otolaryngology - Head and Neck Surgery, IRCCS Azienda Ospedaliero-Universitaria di Bologna, Bologna, Italy; 2 Speech Language Therapist in Otolaryngology and Audiology Unit, Department of Head and Neck Diseases, IRCCS Azienda Ospedaliero-Universitaria di Bologna, Bologna, Italy; 3 Department of Medical and Surgical Sciences (DIMEC), Alma Mater Studiorum University of Bologna, Bologna, Italy

**Keywords:** Voice quality, OPHL, Total laryngectomy, Voice prosthesis

## Abstract

**Objectives:**

The aim of this study is to compare voice outcomes in open partial horizontal laryngectomy vs. total laryngectomy (TL) with voice prosthesis.

**Methods:**

In this retrospective monocentric study patients undergoing OPHL or TL with voice prosthesis were enrolled during the usual oncological follow-up consultations at the Otolaryngology and Audiology Unit of a University Hospital in the period between July 2022 and June 2023. Acoustic analysis (F0, HNR, NHR), maximum phonation time, I-SECEL and INFV0 scale were used to assess voice outcome.

**Results:**

Forty-three patients were enrolled. Voices of patients undergoing LT were better in quality of voice (V0) at INFV0 scale. The scores in I-SECEL and acoustic analysis were comparable.

**Conclusions:**

Voice quality could be slight better in patients undergoing TL with voice prosthesis than those undergoing OPHL.

## Introduction

Laryngeal cancer is the most frequent head and neck cancer, accounting for 60% of cases. Treatment options include surgery and/or chemoradiation, depending on the extent of the disease and patient’s features [1]. Laryngeal surgery can be conservative (partial laryngectomies), aiming to preserve respiratory and phonatory function, or demolitive (total laryngectomy), permanently separating the air and digestive tracts.

Open Partial Horizontal Laryngectomies (OPHLs) require at least one functional crico-arytenoid unit to preserve the organ function. However, this surgery involves a modification of the laryngeal skeleton that significantly alters the anatomy and physiology [2].

Total laryngectomy (TL) results in the loss of laryngeal speech, but various methods, such as voice prosthesis (VP), can be used to restore it. Phonatory valves are inserted into a surgically created fistula between the trachea and pharynx, enabling air passage for speech articulation in the upper vocal tract when the stoma is temporarily closed. VP can be positioned in primary or in a secondary procedure, and requires regular replacements and careful monitoring. However, its high success rate, ranging from 60 to 90%, increases its acceptance among patients and their families [3, 4].

Both OPHL and TL with VP have advantages and disadvantages. OPHL preserves voice, breathing, and swallowing, although complications such as dysphonia, dyspnea, and dysphagia may occur. Dysphagia can be severe and lead to ab ingestis pneumonia, necessitating rehabilitation to improve swallowing function. Dysphonia is often reported as severe, emphasizing the importance of rehabilitation [5–8].

TL, on the other hand, has a significant emotional impact on patients, requiring psychological support and counseling. The surgery results in permanent voice loss and changes in physical appearance, due to the presence of a permanent tracheostoma.

In the existing literature, both approaches have similar oncological outcomes, but the better functional outcome in terms of voice-related quality of life (QoL) and vocal quality remains uncertain [9, 10].

The objective of this study is to compare phonatory functional outcomes and voice-related QoL using objective parameters (F0, MPT, Jitter, Shimmer, HNR, NHR) and subjective scales (I-SECEL and INFV0 scale [11]) in patients who undergo total laryngectomy with phonatory prosthesis placement and those who undergo partial laryngectomies.

## Materials and methods

### Design

Retrospective monocentric study. The study was conducted according to the guidelines of the Declaration of Helsinki and approved by the Ethics Committee of IRCCS Azienda Ospedaliero-Universitaria di Bologna (LARYNX2023, code: CE 279/2023/Oss/AOUBo).

### Participants

A retrospective review was conducted on patients treated by OPHL II-III or TL with VP who had undergone annual oncological and phoniatric follow-up visits at a University Hospital between July 2022 and June 2023. Eligible participants were patients ≥ 18 years old who had undergone OPHL type II-III or TL with VP placement with the ability to produce substitution voice and no evidence of relapse of the disease at last follow up. Two expert speech therapists (MG and AB) had provided voice and swallowing rehabilitation for all cases, both during their hospital stay and after discharge. Patients who were unable to provide informed consent due to age or clinical conditions, as well as those lost to oncological and phoniatric follow-up, were excluded from the study. Patients who still needed artificial nutrition were excluded. In the case of OPHL group, patients with tracheal cannula or tracheostoma at last follow up were excluded from this study.

Data pertaining to the surgical procedure, time between surgery and data collection, pre-operative TNM staging, adjuvant radiotherapy, postoperative complications, and postoperative vocal analysis were collected retrospectively from patients’ medical records, follow-up visits, questionnaires, and voice recordings using the Praat program. For the OPHL group, we specifically considered OPHL type IIa/b or type IIIa/b, arytenoid resection, crico-arytenoid unit (CAU) resection, post-operative radiation treatment. As for the TL with PV group, we also took into account the timing of PV placement, distinguishing between primary and secondary techniques.

Postoperative complications considered in the study included severe dysphagia, pexy detachment, and severe dysphonia.

### Acoustic analysis

Maximum phonation time (MPT) was obtained by asking the patient to sustain the vowel/a/for as long as possible on a single breath. The longest of three attempts was calculated as the MPT. The vowel /a/ and the word /aiuole/ were used to register patient’s voice analysis. PRAAT program was used, and these parameters were analyzed:

- F0: the perceived pitch of a person’s voice. It represents the rate at which the vocal folds vibrate during phonation and is measured in hertz (Hz);
- Harmonic to Noise Ratio (HNR);
- Noise to Harmonic Ratio (NHR).

### Perceptual assessment

A blind perceptual assessment of recorded speech samples (reading task) was conducted by two speech therapists who were trained in substitution voices (MG and AB). The INFVo scale was used, which is a specialized tool designed for perceptual assessment of substitution voices. The scale encompasses five parameters: overall impression (I), intelligible voice (I), unintended additive noise (N), fluency (F), and quality of voicing (Vo). Each parameter is scored on a scale of 0 to 10, with higher scores indicating better perceived voice quality.

### Self-assessment

The Italian Self-Evaluation of Communication Experiences after Laryngeal Cancer (I-SECEL) was designed to assess communication experiences and difficulties faced by individuals who have undergone treatment for laryngeal cancer. The I-SECEL questionnaire is a self-report tool that allows patients to provide their own perspectives and insights regarding their communication experiences. To facilitate patients’ understanding we used the Italian version of the SECEL questionnaire, as validated by Schindler and colleagues [12].

It consists of 35 items, each assessing specific aspects of communication (General, Environment, Attitude). Patients rate their experiences on a 4-point scale. Finally, a score between 0 and 102 is obtained, divided into three subscales. A score below 60, may indicate the need for psychological support to accept the new voice.

### Statistical analysis

Statistical analysis was performed using SPSS 28.0 for Windows (IBM Inc., USA). For discrete variables Chi square test or Fischer’s exact test were used when appropriate. The normality of distribution for continuous variables was assessed with the Shapiro–Wilk test. For continuous variables Mann–Whitney U test or Student’s T test were used respectively for non-normally or normally distributed variables. Association between variables were considered significant for p < 0.05, with confidence interval set at a 95%.

## Results

The study included 43 patients, 27 had undergone OPHL type II or III (OPHL group) and 16 patients had undergone TL with VP placement (TL with VP group). Demographic data of the study population is described in Table 1. The two groups were homogeneous for sex and age.

**Table 1:** Demographics

Descriptive statistics of the main variables concerning patients				
	n	Mean ± SD	Range	P-value
All patients	43			
Gender, male	42			
OPHL group	27			
TL group	15			
Gender, Female	1			
OPHL Group	0			
TL group	1			
Age (years)				
All patients		63.46 ± 11.60	35–83	
OPHL group		61.50 ± 11.07	35–82	NS
TL group		66.87 ± 12.09	47–83	NS
Follow-up (months)				
All patients		22.16 ± 40.21	1.20–233.47	
OPHL group		29.33 ± 10.03	1.20–233.47	NS
TL group		10.06 ± 7.02	1.43–26.37	NS

*NS* non significant

Data regarding surgery and clincial characteristic are described in Table 2. The majority of patients (83.7%, 36/43) underwent laryngeal surgery for squamous cell carcinoma (SCC), the others (13.9%, 6/43) had laryngeal papillomatosis or low-grade malignant myoepithelioma. Regarding the OPHL group, OPHL type IIa was the most frequent surgery performed (77.8%, 21/27), the others underwent OPHL type IIb (18.5%, 5/27) or OPHL type III (3.7%, 1/27). Arytenoid preservation was possible in 16 cases (59.3%), in the remaining 11 cases (40.7%), the arytenoid (72.7%, 8/11) or the cricoarytenoid unit (27.3%, 3/11) were removed. In the TL with VP group, 13/16 patients (81.2%) underwent total laryngectomy while 3/16 patients (18.8%) had total laryngectomy extended to the omolateral piriform sinus. Voice prosthesis was inserted with a primary technique in 14/16 cases (87.5%), with a secondary technique in the remaining 2/16 patients (12.5%). Complications were reported in 4/43 patients (9.3%); 2/27 patients (7.4%) of the OPHL group underwent surgical revision for detachment of the pexy, 2/16 patients (12.5%) of the TL Group had periprosthetic leakage or hematoma in the immediate post-operative period.

**Table 2:** Surgical and clinical data

Descriptive statistics of the main variables concerning surgery and tumours			
	n		
	All patients	OPHL group	TL group
Tumor stage			
pTis	1	1	0
pT1a	4	4	0
pT1b	5	5	0
pT2	11	8	3
pT3	11	4	7
pT4a	5	0	5
Non SCC	6	5	1
Surgery type			
OPHL		27	
IIa		21	
IIb		5	
IIIa		1	
Arytenoid preservation			
Yes		16	
No		11	
Ary removed		8	
CAU removed		3	
TL			16
Extended to Piriform sinus			
Yes			3
No			13
VP			
Primary			14
Secondary			2
Complications	4	2	2
Post-operative RT			
Yes	12	2	10
No	31	25	6

Acoustic analysis data are reported in Table 3. Mean MPT of the TL group was higher than mean MPT of the OPHL group but this result did not reach statistical significance. No statistically significant difference was observed between the two groups in terms of F0, NHR, HNR and MPT.

**Table 3:** Acoustic analysis

Acoustic analysis and maximum phonation time			
	OPHL Group (n = 27)	TL Group (n = 16)	P-value
	Mean ± SD	Mean ± SD	
A			
F0	159.23 ± 116.80	221.75 ± 184.77	0.843
NHR	0.61 ± 0.22	0.72 ± 0.16	0.234
HNR	3.06 ± 2.82	1.87 ± 1.05	0.327
MPT	8.18 ± 5.95	10.95 ± 6.10	0.088
AIUOLE			
F0	149.20 ± 127.60	177.85 ± 125.47	0.295
NHR	0.60 ± 0.17	0.58 ± 0.15	0.581
HNR	2.89 ± 1.68	3.32 ± 1.78	0.615

Experienced speech therapists conducted the perceptual assessment of voice using the INFV0 scale (Table 4). TL group had a significantly better outcome in terms of Quality of Voicing (Vo) vs. OPHL group (mean score: 8.25 ± 1.61 vs. 6.84 ± 2.34). No statistically significant difference was observed between the two groups in the other parameters of the INFV0 scale.

**Table 4:** Perceptual assessment

INFVo scale: average scores, standard deviations and p values			
	OPHL Group (n = 27)	TL Group (n = 16)	P-value
	Mean ± SD	Mean ± SD	
Overall impression (I)	7.40 ± 1.98	8.31 ± 1.85	0.124
intelligibility (I)	7.68 ± 1.97	8.43 ± 1.82	0.185
Noise (N)	7.72 ± 2.34	8.00 ± 2.56	0.511
Fluency (F)	8.60 ± 1.41	8.94 ± 1.57	0.274
Voicing (Vo)	6.84 ± 2.34	8.25 ± 1.61	0.046

Results from patients self-evaluation questionnaire (I-SECEL) are reported in Table 5. Mean total score between the groups were similar (mean OPHL group: 51.42 ± 16.00; mean TL group: 53.67 ± 17.77). No statistically significant difference was observed in any of the subscale of the I-SECEL questionnaire.

**Table 5:** Patient’s self evaluation

I-SECEL questionnaire			
	OPHL group (n = 27)	TL group (n = 16)	P-value
	Mean ± SD	Mean ± SD	
Total score	51.42 ± 16.00	53.67 ± 17.77	0.860
General subscale	10.30 ± 2.65	10.20 ± 2.34	0.881
Environment subscale	28.73 ± 7.57	31.07 ± 10.78	0.524
Attitude subscale	12.39 ± 10.75	12.40 ± 8.75	0.881

## Discussion

Current literature presents very few studies comparing voice outcome between partial and total laryngectomy [9]. Functional results, such as the risk of dysphagia and dysphonia after these treatments should be well illustrated to the patients before surgery. Overall, the subjective and objective voice outcome of this study showed satisfactory results for both groups. In the acoustic analysis, the maximum phonation time of the TL group was longer than in the OPHL group, despite no statistically significant difference. The neoglottis of patients who undergo partial laryngectomy does not always allow complete glottic closure, and air leaks could be present; this could explain the shorter MPT in the OPHL group. However, this result need to be confirmed with a larger cohort of patient. Expert-rated voice quality results using the INFV0 scale demonstrated that the voice parameter quality of voicing (V0) was significantly higher in the LT group. The V0 parameter indicates if voicing is voiced or unvoiced. This result falls in line with the previous study conducted by D’Alatri et al. [9]. In their study not only the quality of voicing (V0) but also the overall voice quality parameter (I) was significantly higher in the LT group. In our case, the overall voice quality was higher in the OPHL group but it did not reach a statistical significance difference compared to the TL group. These results may be explained by a different anatomy of the vibratory structure that results from the two surgical techniques. In partial laryngectomy the arytenoids, the base of tongue and the epiglottis are responsible for the vibration source for voice production. On the other hand in total laryngectomies the pharyngo-oesophageal segment is responsible for voice production. These results, together with the one of the previous published study [9], show that the neoglottis of partial laryngectomy may not be as effective as that of the pharyngo-oesophageal segment after TL. However no differences were found in the speech related parameters (N and F). Regarding total laryngectomy it would be of interest to investigate voice differences between patients treated with surgery alone and patients treated with adjuvant radiotherapy. Finally, self-related voice assessment results (I-SECEL questionnaire) did not show significant differences between the two groups. These results fall in line with the current literature [9, 13] in which, despite different vocal characteristics, patients undergoing total laryngectomy and partial laryngectomy reported a similar voice-related quality of life.

Our study has some limitations, including its retrospective nature, which may introduce biases. Firstly, only univariate analysis has been performed reflecting the parameters’ distribution in our patients’ cohort not taking into account how independent variables influence each other. Additionally, considering the relative limited number of patients we decided not to perform a subgroup statistical analysis regarding the type of surgical resection (e.g. OPHLIIa vs OPHLIIb).

Future multicentric studies with a larger sample size are planned to assess the correlation between surgical resection extent and voice outcomes and to analyse the patterns and correlations between voice parameters through a comprehensive multivariate analysis. Finally, the use of a patient subjective evaluation questionnaire (I-SECEL) is a key parameter for a comprehensive voice quality evaluation. However, it should be kept in mind that the I-SECEL questionnaire showed low consistency in five out of thirty-five items in its validation study.

## Conclusion

Despite objective and subjective voice results in favor of total laryngectomy, the burden of a permanent tracheostoma in patients who undergo total laryngectomy and its consequences should be considered during patient counselling. On the other side, OPHL requires longer rehabilitation periods in order to obtain acceptable voice outcomes. Hopefully, this study can help the clinician and the patient to have a wider overview of the functional results of these surgeries improving the pre-operatory counselling.
